# Shelf-Life Extension of Fc-Fused Single Chain Fragment Variable Antibodies by Lyophilization

**DOI:** 10.3389/fcimb.2021.717689

**Published:** 2021-11-15

**Authors:** Kai-Thomas Schneider, Toni Kirmann, Esther Veronika Wenzel, Jan-Hendrik Grosch, Saskia Polten, Doris Meier, Marlies Becker, Paul Matejtschuk, Michael Hust, Giulio Russo, Stefan Dübel

**Affiliations:** ^1^ Department of Biotechnology, Technische Universität Braunschweig, Braunschweig, Germany; ^2^ Abcalis GmbH, Braunschweig, Germany; ^3^ Institute of Biochemical Engineering, Technische Universität Braunschweig, Braunschweig, Germany; ^4^ Center of Pharmaceutical Engineering, Technische Universität Braunschweig, Braunschweig, Germany; ^5^ Standardisation Science, National Institute for Biological Standards & Control (NIBSC), Hertfordshire, United Kingdom

**Keywords:** phage display, recombinant antibody, lyophilization, freeze-drying, antibody stability, antibody formatting, IgG, scFv-Fc

## Abstract

Generation of sequence defined antibodies from universal libraries by phage display has been established over the past three decades as a robust method to cope with the increasing market demand in therapy, diagnostics and research. For applications requiring the bivalent antigen binding and an Fc part for detection, phage display generated single chain Fv (scFv) antibody fragments can rapidly be genetically fused to the Fc moiety of an IgG for the production in eukaryotic cells of antibodies with IgG-like properties. In contrast to conversion of scFv into IgG format, the conversion to scFv-Fc requires only a single cloning step, and provides significantly higher yields in transient cell culture production than IgG. ScFv-Fcs can be effective as neutralizing antibodies *in vivo* against a panel of pathogens and toxins. However, different scFv fragments are more heterologous in respect of stability than Fab fragments. While some scFv fragments can be made extremely stable, this may change due to few mutations, and is not predictable from the sequence of a newly selected antibody. To mitigate the necessity to assess the stability for every scFv-Fc antibody, we developed a generic lyophilization protocol to improve their shelf life. We compared long-term stability and binding activity of phage display-derived antibodies in the scFv-Fc and IgG format, either stored in liquid or lyophilized state. Conversion of scFv-Fcs into the full IgG format reduced protein degradation and aggregation, but in some cases compromised binding activity. Comparably to IgG conversion, lyophilization of scFv-Fc resulted in the preservation of the antibodies’ initial properties after storage, without any drop in affinity for any of the tested antibody clones.

## Introduction

Antibodies play a major role in modern medicine and serve as indispensable tools in the field of research, diagnostics and therapy (Borrebaeck, 2000; Rhodes and Trimmer, 2006; Kaplon and Reichert, 2021). With an approximated market value of US$ 137 – 200 billion in 2022 monoclonal antibodies are considered the fastest growing class of therapeutic proteins (Ecker et al., 2015; Grilo and Mantalaris, 2019). Between 1997 and 2013, 34 antibody therapeutics have been first approved by either EMA and/or FDA. Within only the last ~7 years (March 2021) this number has tripled, with even more currently being in clinical trials (Kaplon and Reichert, 2021).

Over the past decades, *in vitro* selection systems, most prominently antibody phage display, have gained widespread significance (Valldorf et al., 2021). This robust method allows the rapid generation of sequence-defined human antibodies from naïve, immune or synthetic libraries and has therefore become an increasingly meaningful tool in the discovery of antibodies against targets of all kinds (McCafferty et al., 1990; Barbas et al., 1991; Breitling et al., 1991; Kügler et al., 2015; Alfaleh et al., 2020; Wenzel et al., 2020).

The modular nature of antibodies enables a broad spectrum of possibilities to generate novel recombinant molecules with tailored properties to the final application. ScFv fragments obtained from common phage display techniques are the smallest binding part of a human immunoglobulin retaining the full specificity, comprising only the variable parts of heavy (VH) and light chain (VL) fused by a polypeptide linker (Holliger and Hudson, 2005; Frenzel et al., 2013). Due to their small size (~30 kDa) scFv antibodies are known for their higher tissue penetration and exhibit rapid serum clearance by renal filtration and lack of recycling mediated by FcRn binding, which makes this format feasible for applications like radioimmunotargeting (Milenic, 2000; Thurber et al., 2008; Li et al., 2016).

However, for most therapeutic approaches Fc-mediated effector functions such as prolonged serum half-life, antibody-dependent cellular cytotoxicity and activation of the complement system are crucial (Lee et al., 2008; Jazayeri and Carroll, 2008). Thus, the vast majority of antibodies that recently have been approved by EMA or FDA and those in late-stage clinical studies are still full-length IgGs (Kaplon and Reichert, 2021). While final therapeutic antibody drugs mainly rely on the full IgG format, conversion of promising candidates from the scFv format into IgG has to be performed in two independent cloning steps, one per each chain, making it a rather laborious process (Powers et al., 2001). The Fc moiety can also be genetically fused to an scFv *via* a single cloning step and without need for DNA amplification (Powers et al., 2001; Schirrmann and Büssow, 2010). Consequently, during their development, the screening of large numbers of candidates in functional *in vitro* or *in vivo* assays requiring bivalency and/or Fc functions can then be accelerated by using the simpler one step scFv to scFv-Fc conversion (Bertoglio et al., 2021). Further, scFv-Fcs are often produced with much higher yields in transient cell culture systems (see also **Table 3**), making this format more amenable to HTS production of a large numbers of clones. Using Fc moieties from different species can facilitate assays in non-human models, or it can enable multiplex assays (Moutel et al., 2009). Some scFv-Fcs could directly be used for diagnostics and therapy (Kenanova et al., 2005; Wan et al., 2013; Sokolowska-Wedzina et al., 2017; Bernhard et al., 2019; Hay et al., 2020).

As of today, a number of scFvs are used for therapeutic applications, especially in CAR-T (Guedan et al., 2019) or bispecific antibody constructs (Brinkmann and Kontermann, 2017). ScFv fragments lack the CH1/CL immunoglobulin domain. It has been shown that CH1/CL interaction is responsible for most of the Fab fragment stability due to their interchain disulfide bond and not just by domain association to the variable parts (Röthlisberger et al., 2005), a matter that consequently impacts also scFv-Fc antibodies. ScFv molecules are kept together by a flexible linker, allowing some of them to oscillate between an open and a closed state. This can lead to diabody formation and can be strongly influenced by the linker length, antigen presence, pH, or concentration, affecting folding or oligomerization (Arndt et al., 1998; Schmiedl et al., 2000). Different approaches have been proposed to improve scFv stability by forcing a VH/VL pair close state with the introduction of a non-native disulfide bond between the two variable parts (Brinkmann et al., 1993; Trivedi et al., 2009; Zhao et al., 2010; Cai et al., 2016), cyclization (Yamauchi et al., 2019), or by means of mutagenesis of residues affecting the overall molecule propensity to aggregate (Zhang et al., 2015; Austerberry et al., 2019). However, these methods require considerable effort and are for these reasons typically applied only at the end of the antibody lead candidate selection process.

During storage, proteins are exposed to a variety of physicochemical stresses that can impair their integrity and stability dependent on the intrinsic sequence and conformational nature of a protein (Manning et al., 2010; Obrezanova et al., 2015). Among other factors, degradation of human monoclonal antibodies occurs typically *via* Asn deamidation, N-terminal Glu cyclization forming pyroglutamate and fragmentation within the hinge region with a rate dependent on pH value und temperature (Cordoba et al., 2005; Liu et al., 2006; Pace et al., 2013). Chemical degradation is subsequently interrelated with a higher risk of aggregation. Moreover, unfolding and protein-protein association as well as extreme pH values and shifts have to be taken into account as sources of aggregates (Manning et al., 2010). Since air-liquid and solid-liquid interfaces contribute to the formation of aggregates, too, it is important to state that not only chemical processes and environmental factors drive protein destabilization, but also sample handling and storage conditions (Wang et al., 2010).

In general, proteins stored in solid form are more stable than liquid formulations, because in the absence of water hydrolysis reactions are decelerated. Hence, shelf-life of proteins can be extended by means of drying (Harris et al., 2004). Freeze-drying is a comparatively gentle method for drying products that is based on the principle of sublimation. Therefore, removal of water can be performed while circumventing exposure of proteins to critical high temperatures (Franks, 1998). Moreover, freeze-drying results in products that can easily be rehydrated. Thus, the process is also referred to as lyophilization (ancient Greek: *λύω*/leo “to dissolve” and *φιλέω*/phileo “to love”) (Varshney and Singh, 2015). For the reasons mentioned above, despite substantial costs, lyophilization is the method of choice for preservation of sensitive biopharmaceuticals that are prone to loss of stability when stored in solution. About 50% of the currently marketed biopharmaceuticals are lyophilized, including blockbuster monoclonal antibodies such as Infliximab or Trastuzumab (Schwegman et al., 2005; Bjelošević et al., 2020). Indeed, non-native aggregation and degradation are known to not only possibly vanish a protein therapeutic’s desired biological function, but they can also lead to adverse immunogenicity (Xu et al., 2019).

Briefly, a lyophilization cycle can be divided into three phases. First, the sample will be frozen. This step is critical in respect of protein destabilization. Different components of the formulation can sequentially crystallize, which may result in harmful pH shifts or concentration peaks. This can lead to increased protein-protein interactions resulting in a higher likelihood of aggregation and increased ionic strength (Franks, 1998; Tang and Pikal, 2004; Kasper and Friess, 2011). Both can be counteracted by using a suitable buffer system and cryoprotectant excipients, mainly non-reducing disaccharides such as sucrose or trehalose that protect the protein during freezing *via* mechanisms like vitrification and preferential exclusion. Formation of phase interfaces can additionally be reduced by the use of polysorbate 20 or other non-ionic surfactants (Crowe et al., 1996; Roos, 1997; Schwegman et al., 2005; Bhatnagar et al., 2007; Emami et al., 2018). After freezing, sublimation of crystalline water will be initiated by setting a vacuum and subsequently slightly increasing the temperature. This step is called primary drying, followed by secondary drying. Here, the temperature will be further increased in order to remove residual amorphous water by diffusion (Franks, 1998; Colandene et al., 2007). Since hydrogen bonds can’t be built properly, dehydration has a huge impact on in particular sensitive proteins’ secondary structure. Disaccharides can serve as water replacement in order to maintain the native protein’s structure (Prestrelski et al., 1993). Furthermore, additional excipients like mannitol can be used as crystalline bulking agents that facilitate the formation of an elegant lyophilization cake appearance (Horn and Friess, 2018).

In this study, we describe a novel scFv-Fc lyophilization protocol able to preserve the properties of freshly produced scFv-Fc antibodies over at least six months in terms of molecular mass (MM) distribution and binding activity. The protocol was also validated for lyophilization of IgGs. By comparing the activity of the same antibody in scFv-Fc or IgG format, we found that not all scFv antibodies tolerate IgG conversion, highlighting the need for a suitable alternative bivalent antibody format for these clones’ characterization.

## Material and Methods

### Cloning, Expression, and Purification of scFv-Fc and IgG Antibodies

Conversion of phage display derived scFv fragments into scFv-Fc format with either human or murine Fc parts was performed by subcloning scFv encoding DNA into the mammalian cell production vectors pCSE2.6-hIgG1-Fc or pCSE2.6-mIgG2a-Fc (Jäger et al., 2013) using NcoI/NotI (New England Biolabs) restriction. In order to generate mouse IgG antibodies VH and VL were cloned into pCSEHm2a.2 (heavy chain) and pCSLmK.2/pCSLmL3.2 (kappa/lambda light chain). The vectors are mouse variants of pCSH1c or pCSL3I, respectively (Steinwand et al., 2014). Cloning was performed *via Golden gate assembly* approach (Engler et al., 2008) with Esp3I endonuclease (New England Biolabs) and T4 DNA ligase (Promega). For this purpose, the Esp3I restriction sites were introduced into the desired VH and VL by PCR using Q5 High-Fidelity DNA Polymerase (New England Biolabs). The original scFv genes served as template DNA.

Production of scFv-Fc and IgG antibodies was done in Expi293F (Thermo Fisher Scientific) or HEK293-6E suspension cells (Jäger et al., 2013). Cells were transfected using polyethylenimine (PEI, Polysciences) and cultured at 37°C, 110 rpm and 5% CO_2_ in serum-free Gibco™ FreeStyle™ F17 expression media (Thermo Fisher Scientific) supplemented with 7.5 mM Glutamine (Biochrom) and 0.1% Pluronic F68 (PAN Biotech). After 48 hours the culture volumes were doubled with either fresh F17 medium for Expi293F or HEK TF (Xell) supplemented with 8 mM L-Glutamine and 1/10 volume HEK FS (Xell) for HEK293-6E cells. Five days later the cultures were harvested followed by purification of the obtained antibodies *via* Protein A affinity chromatography using MabSelect™ SuRe™ resin (Cytiva). After purification the elution buffer was exchanged for 1 x PBS using Zeba™ Spin Desalting Columns (Thermo Fisher Scientific) or dialysis. Antibodies were stored at -80°C until further use.

### Sample Preparation and Lyophilization Procedure

TUN219-2C1-hFc, a scFv-human IgG1-Fc fusion (Russo et al., submitted), was differently formulated in order to screen for beneficial combinations of buffer and excipients for lyophilization. Sample preparation consisted in performing buffer exchange from 1 x PBS using Zeba™ Spin Desalting Columns (Thermo Fisher Scientific). Afterwards, 1 or 2% (w/v) trehalose or sucrose or the equivalent amount of water were added and the antibody’s concentration was adjusted to 0.5 mg/mL. In the protocol validation and IgG comparison section only the best tested formulation containing 10 mM sodium phosphate buffer (pH 7.4) with 2% (w/v) trehalose was used.

Nunc MaxiSorp™ flat-bottom multiwell strips (Thermo Fisher Scientific) were used in the formulation screening section and contained 105 µL per well with triplicates of each formulation distributed on different strips. The strips were randomly placed on the precooled (4°C) shelf of a Beta 2-8 LSCplus lyophilization machine (Martin Christ) and loosely closed with TPE lyophilization caps (Micronic). Lyophilization was done by single chamber operation. Freezing of samples was carried out inside the chamber by decreasing the shelf-temperature to - 35°C within 1 h and keeping this temperature for additional 5 h. Afterwards, sublimation was initiated by setting a vacuum of 0.1 mbar. Primary drying was then performed for 12 h. Following this, the shelf temperature was increased to 20°C within 5 h while the vacuum was furthermore decreased to 0.08 mbar in order to initiate secondary drying. This final shelf temperature and pressure were kept for additional 2 h. After the lyophilization cycle was completed the chamber was flooded with nitrogen until ambient pressure was reached before the wells were tightly closed. Lyophilizates were reconstituted in 250 µL ultrapure water for functional analysis.

Lyophilization in 2 mL crimp top glass vials with a diameter of 11 or 15 mm (Phenomenex or Wheaton Industries, respectively) was performed using 189 µL or 315 µL antibody solution, respectively. The latter sample size was used in the initial formulation screening section. The vials were randomly positioned on the shelf and loosely closed using lyophilization caps (189 µL) or rubber stoppers (Wheaton Industries) (315 µL). All samples with 189 µL volume were lyophilized using the Alpha 2-4 LSCplus (Martin Christ) with an internal freezing step in which the temperature was decreased to - 65°C within 3 h. Samples with 315 µL volume were frozen externally at - 80°C over night on the machine’s demountable shelf before using the Beta 2-8 LSCplus. Primary drying was performed by setting a vacuum of 0.1 mbar, keeping the shelf temperature at - 50°C for 30 min and finally increasing it to - 35°C within 1 h. This condition was kept for 36 h followed by decreasing the vacuum to 0.08 mbar and increasing the temperature to 20°C within 16 h in order to initiate secondary drying. After additional 16 h the procedure was stopped and the chamber was either flooded with air (Alpha 2-4 LSCplus) or nitrogen (Beta 2-8 LSCplus), before the vials were tightly closed. In case lyophilization caps were used, the rims of the closed vial lids were additionally sealed with parafilm. Reconstitution was performed with volumes of ultrapure water identical to the pre-lyophilization volumes.

### Storage Conditions

The impact of time and temperature on lyophilized scFv-Fc and IgG antibodies under different storage conditions was compared with equivalent aqueous samples in 1 x PBS. For this purpose, up to three replicates for each storage condition were lyophilized as described in the same cycle. Additionally, up to three aliquots of the same antibody production and purification were diluted to 0.5 mg/mL in 1 x PBS in PCR tubes or 2 mL screw cap micro tubes. The tubes were then additionally sealed with parafilm. Lyophilized and liquid samples were stored at 4°C or RT for up to 6 month or at 45°C for 2 days. Samples that have directly been stored at - 80°C after purification or subsequent lyophilization were considered as 0 days of storage time.

### Selection of Formulations Based on Cake Appearance

Per each condition, three individual samples were inspected for successful lyophilization based on the lyophilizate’s macroscopic appearance. Exclusion criteria were meltbacks or major cake collapse as well as unusually long reconstitution times, but not shrinkage of a formed cake. The formulation was considered unsuccessful if at least two out of three samples met an exclusion criterion.

### Antibody Titration ELISA

In order to compare the binding activity of scFv-Fc and IgG antibodies, an enzyme-linked immunosorbent assay (ELISA) was performed. 96-well MTPs (High Bind, Corning) were coated overnight at 4°C with 100 ng/well of the corresponding antigen in 1 x PBS. Additionally, unspecific binding was tested on unrelated proteins. The next day, residual liquid was removed followed by blocking the wells with 350 µL 2% M-PBST (2% (w/v) milk powder dissolved in 1 x PBS; 0.05% Tween20) for 1 h at RT. Afterwards, the blocked ELISA plates were washed three times with H_2_O and 0.05 % Tween20 and antibodies were titrated from 1 µg/mL to 3.16 pg/mL or 10 µg/mL to 500 pg/mL. Binding took place for 1 h at RT followed by three washing cycles with H_2_O and 0.05 % Tween20. Hereafter, bound antibodies were detected for 1 h at RT with 100 µL horseradish peroxidase (HRP) conjugated goat anti-hIgG (A0170, Sigma) or goat anti-mIgG serum (A0168, Sigma) diluted 1:70,000 or 1:42,000, respectively, in 2% M-PBST. Next, unbound antibodies were washed away three times with H_2_O and 0.05% Tween20 before 100 µL freshly prepared tetramethylbenzidine (TMB) substrate (20 parts TMB solution A (30 mM potassium citrate; 1% (w/v) citric acid (pH 4.1)) and 1 part TMB solution B (10 mM TMB; 10% (v/v) acetone; 90% (v/v) ethanol; 80 mM H_2_O_2_ (30%)) was dispensed to each well and incubated at RT. After the color reaction was stopped by adding 100 µL 1 N sulfuric acid, the absorbance at 450 nm was measured with a reference at 620 nm using an ELISA plate reader (Epoch, BioTek). EC_50_ values were calculated with OriginPro (versions 2016, 2018, 2020 and 2021, OriginLab) using Logistic5 curve fit.

### Size Exclusion Chromatography

Antibody MM distributions dependent on different storage conditions were investigated by either size-exclusion high performance liquid chromatography (SE-HPLC) or size exclusion chromatography (SEC). To avoid clogging of the column, each sample was centrifuged at 3200 x g for 30 min and subsequently filtered (0.2 µM). For analysis *via* SE-HPLC, the separation was performed in 150 mM sodium phosphate buffer (pH 7.0) using an AdvanceBio SEC 300 column (Agilent Technologies) with a separation limit of 5 – 1250 kDa. Prior to injection the samples were diluted in 400 µL running buffer to a final concentration of 50 µg/mL. Per run a volume of 20 µL was injected and separated with a flow rate of 0.35 mL/min using Clarity (DataApex) for operation. Chromatograms were further analyzed with Origin. The separation *via* SEC was performed in 50 mM sodium phosphate buffer with 150 mM NaCl (pH 7.0) using a Superdex200 Increase 10/300 GL column (Cytiva) with a separation limit of 10 - 600 kDa. Antibodies were injected onto the column in quantities ranging from 10 to 50 μg. The flow rate was set to either 0.5 or 0.75 mL/min. For the operation of the SEC systems and for data export the software UNICORN 5.10 or UNICORN 7.0 was used. Afterwards the chromatograms were evaluated using the OriginPro software (versions 2016, 2018, 2020 and 2021). Both columns were calibrated according to the manufacturer’s instructions using the “Gel Filtration Markers for Protein Molecular Weights 12,000 - 200,000” kit (Sigma-Aldrich).

## Results

### Formulation Screening in MTPs

Proteins are exposed to a variety of mechanical and physicochemical stresses during a freeze-drying process. Therefore, an initial selection of 55 formulations was screened for their capability to protect a monoclonal anti-c-Myc tag scFv-Fc antibody (TUN219-2C1-hFc), which was selected for its non-optimal stability, during lyophilization and subsequent storage. In order to allow a sufficient number of replicates, this lyophilization was performed in microplate strips.

**Figure 1:**
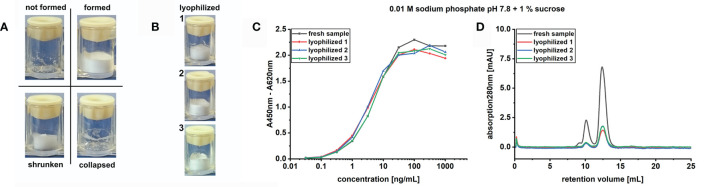
Lyophilization protocol screening parameters. **(A)** Cake appearance categorization based on optical evaluation. Formed or formed and shrunken cakes are assigned a “+” in **Table 1** or an “x” when difficult to reconstitute, not-formed or collapsed cakes receive a “-” in the same table. **(B)** Exemplification of correctly formed cakes in independent sample triplicates. Comparison of fresh *versus* lyophilized sample in **(C)** indirect ELISA, to study the molecules binding activity, or **(D)** in SEC, for the determination of the molecular mass distribution. SEC was performed using 50 µg fresh or 10 µg lyophilized antibody.

Lyophilized samples were first compared based on their macroscopic appearance (**Figure 1A**). All formulations containing 0.01 M sodium or potassium phosphate buffer in combination with trehalose or sucrose as cryoprotectant led to the formation of lyophilization cakes, which in a few cases had a slightly shrunken appearance, whereas the 2% solutions were basically favorable in terms of cake uniformity. Without the addition of any excipients, only formulations containing 0.1 M sodium phosphate or 1 x PBS resulted in adequate lyophilization cakes. The use of 0.1 M potassium phosphate or Tris buffers resulted in poor to no cake formation independently from the addition of either trehalose or sucrose (**Table 1**).

**Table 1:** Buffer formulation screening based on lyophilized cake optical appearance immediately after freeze-drying in microtiter plates.

	Acceptable cake apperance														
	no excipient			1 % trehalose			2 % trehalose			1 % sucrose			2 % sucrose		
1 x PBS pH 7.4	+	+	–	–	–	–	–	–	–	–	–	–	–	–	–
0.1 M potassium phosphate pH 7.0	–	–	–	–	–	–	–	–	–	–	–	–	–	–	–
0.01 M potassium phosphate pH 7.0	–	–	–	+	+	+	+	+	+	+	–	+	+	+	+
0.1 M potassium phosphate pH 7.4	x	x	–	–	–	–	–	–	–	+	+	+	–	–	–
0.01 M potassium phosphate pH 7.4	–	–	–	+	–	+	+	+	+	+	+	+	+	+	+
0.1 M sodium phosphate pH 7.4	+	+	–	–	+	–	–	–	+	+	+	+	–	–	+
0.01 M sodium phosphate pH 7.4	–	–	+	+	–	+	+	+	+	+	+	+	+	+	+
0.1 M sodium phosphate pH 7.8	+	+	+	+	+	–	+	+	+	x	x	x	+	+	+
0.01 M sodium phosphate pH 7.8	+	–	–	+	+	+	+	+	+	+	+	+	+	+	+
0.1 M Tris-HCl pH 6.8	–	+	–	–	–	–	–	–	–	–	–	–	–	–	–
0.01 M Tris-HCl pH 6.8	–	–	–	+	–	–	x	–	x	–	–	–	+	+	+

Formed or formed but shrunken cakes are assigned a “+” or an “x” when difficult to reconstitute, not-formed or collapsed cakes receive a “-”. Illustration of the corresponding optical appearances is given in **Figure 1**. Three values per formulation are resultant from three lyophilized products.

For those buffer formulations where a cake formed and could be reconstituted, the binding activity of TUN219-2C1-hFc prior and post sample preparation and lyophilization was compared in antigen ELISA (**Figure 1C**). The sigmoidal binding curves describing concentration-dependent antigen binding of each lyophilized sample with EC_50_ values in the range of 1.1 ± 0.1 ng/mL (0.01 M sodium phosphate pH 7.4 with 1% trehalose) to 4.6 ± 0.1 ng/mL (0.1 M sodium phosphate pH 7.8 without excipient) overlapped with the one of fresh antibody (EC_50_ = 3.9 ± 1.1 ng/mL) (**Supplementary Table 1**). In addition, no increase in stickiness was detected due to lyophilization, as proven by the absence of binding on unrelated antigen (data not shown). While binding activity in ELISA did not show detectable differences among the tested buffer formulations for lyophilization, this cannot exclude differences in the protein’s physical state.

To investigate whether or how the antibody stability profile was affected by the presence of specific buffers and excipients during lyophilization, we analyzed each sample in size-exclusion chromatography (SEC) including the freshly produced TUN219-2C1-hFc as reference (**Figure 1D**). The chromatogram showed the presence of distinctive peaks, with the first and most prominent occurring at 12.4 mL. The corresponding molecular mass (MM) was calculated to ~86 kDa, despite the MM was of 109 kDa according to its protein sequence (two 54.5 kDa monomers). Relative MM and the absence of degradation were assessed in SDS-PAGE and coomassie blue staining of scFv-Fc separated under reducing conditions, which showed a double band ~55 kDa corresponding to glycosylated and unglycosylated protein (data not shown). In SEC, a molecule’s retention is based on its hydrodynamic radius and interaction with the column material. Thus, the calculated size may not be equivalent with its actual MM (Ghose et al., 2007; Philo, 2009). Thereof, it can be assumed that this peak represents the flexible scFv-Fc fusion protein monomer. A second peak with a shoulder occurring at smaller retention volumes represents high molecular mass species (HMMS). Additionally, a peak occurred at a retention volume of 0.29 mL, but the comparison with a sample run under injection of pure water indicated that this is a protein-independent injection peak (data not shown). The chromatograms of the lyophilized scFv-Fcs showed only slight deviations from the fresh sample (**Figure 1D**). For all formulations examined, peaks appeared for monomers and HMMS but these were different with respect to proportions among each other and from the control.

The peak areas were calculated and summarized (**Supplementary Table 1**). The monomer fraction of the fresh reference sample was 76.9 ± 0.6%. Freeze-drying using potassium phosphate buffers generally resulted in slightly reduced monomer content. The same was observed for Tris-HCl buffered and 1 x PBS formulations. Lyophilization in sodium phosphate buffered formulations, on the other hand, tended to result in most cases in even higher monomer contents than that of the control with a maximum of 82.1 ± 1.7% for 0.01 M sodium phosphate pH 7.4 with 2% trehalose. However, 0.1 M sodium phosphate pH 7.4 without excipient showed the lowest monomer portion among all formulations (73.9 ± 2.9%). Finally, a significant influence of the pH value and the cryoprotectant substances with regard to aggregation and fragmentation of the antibody could not be derived from the results.

Overall, sodium phosphate proved to be the most suitable buffer to preserve TUN219-2C1-hFc stability during lyophilization. Other buffer systems, as well as 0.1 M sodium phosphate pH 7.4 without added sugar, were therefore excluded from further validation work.

### Shelf-Life Assessment of Preselected Formulations in Glass Vials

Since glassware containers such as vials and ampullae are favored for freeze-drying, long-term stability tests with the formulations selected in the previous screening were performed using 2 mL (15 mm) glass vials. Freeze-drying with the use of a cycle adapted to the higher vial volume was performed in triplicates and followed by sample storage for thirty days at RT or - 80°C.

In line with the results from the initial lyophilization buffer screening, each tested formulation resulted in the formation of cakes with correct appearances in glass vials. All cakes stored at –80°C for 30 days showed no visual alteration of their macroscopic structure independently from the formulation adopted for lyophilization. Only after 30 days of storage at RT, it was possible to discriminate between formulations resulting in the formation of a durable product based on cake appearance (**Table 2**). Remarkably, only 2 of 18 samples containing sucrose withstand storage at RT, while in the presence of 1% trehalose the formed cake collapsed over time or the antibody was difficult to reconstitute. At 0.01 M concentration, sodium phosphate buffer was confirmed to work with the addition of 2% trehalose and undergo the storage period optically unchanged. In contrast, we observed that 0.1 M sodium phosphate buffers only in absence of sugar resulted in durable cakes. In the presence of sugar, however, every cake lost its structure over time.

**Table 2:** Buffer formulation screening based on lyophilized cake optical appearance after 30 days post lyophilization in glass vials.

	Acceptable cake appearance																													
	No excipient						1 % trehalose						2 % trehalose						1 % sucrose						2 % sucrose					
	-80°C			RT			-80°C			RT			-80°C			RT			-80°C			RT			-80°C			RT		
0.1 M sodium phosphate pH 7.4																			+	+	+	–	–	+						
0.01 M sodium phosphate pH 7.4							+	+	+	x	x	x	+	+	+	+	+	+	+	+	+	–	–	–	+	+	+	**-**	**-**	**-**
0.1 M sodium phosphate pH 7.8	+	+	+	+	+	+	+	+	+	–	–	–	+	+	+	+	–	–							+	+	+	**-**	**-**	**+**
0.01 M sodium phosphate pH 7.8							+	+	+	x	–	x	+	+	+	+	+	+	+	+	+	–	–	–	+	+	+	**-**	**-**	**-**

Formed or formed but shrunken cakes are assigned a “+” or an “x” when difficult to reconstitute, not-formed or collapsed cakes receive a “-”. Six values per formulation are resultant from three lyophilized products each stored at - 80°C and RT.

**Figure 2:**
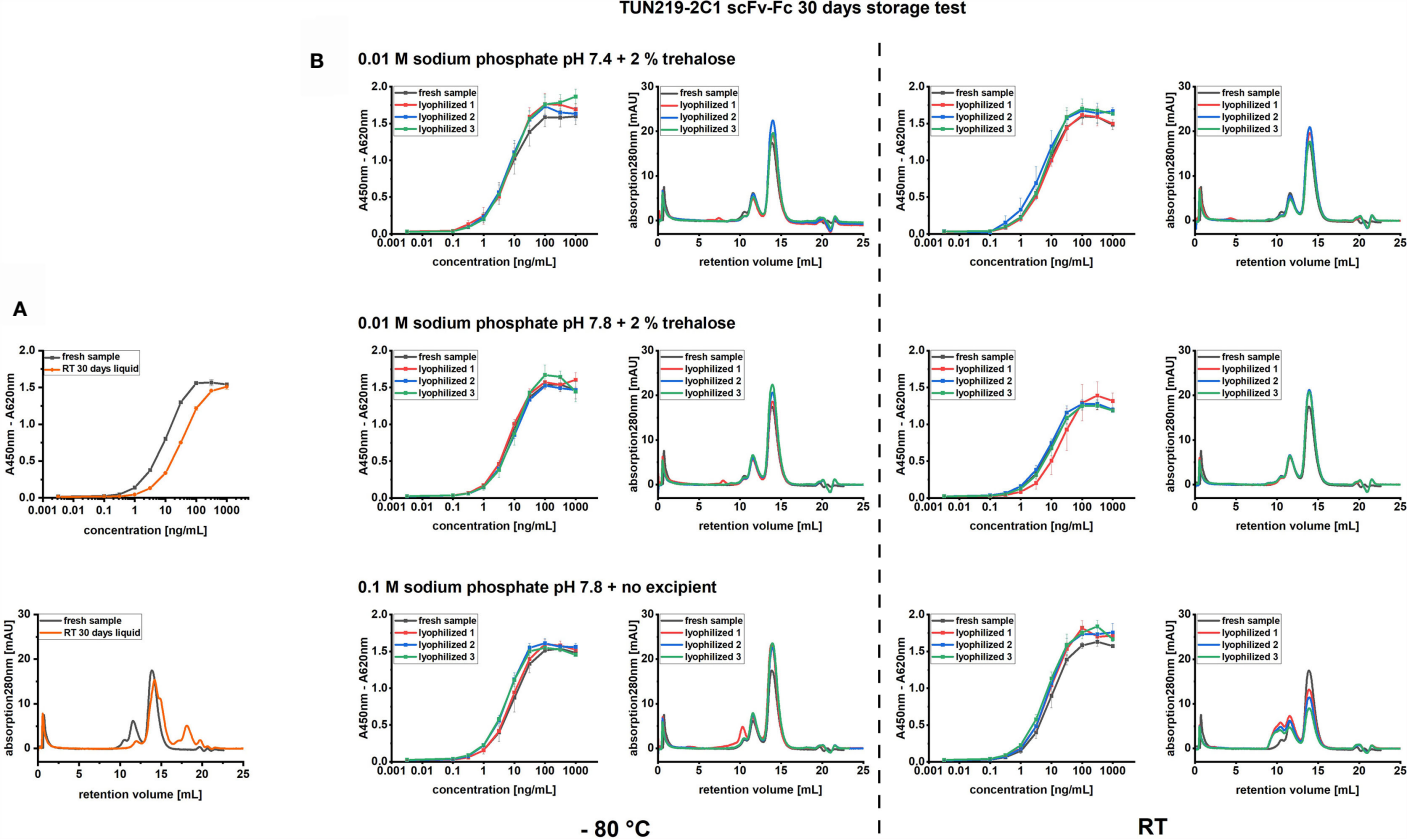
Model antibody storage test. **(A)** TUN219-2C1-hFc antibody after storage for 30 days at RT in 1 x PBS solution was compared with the fresh antibody in SEC and indirect ELISA, where 100 ng immobilized antigen were incubated with TUN219-2C1-hFc in a concentration range spanning from 3.16 pg/mL to 1 µg/mL. **(B)** Differently formulated lyophilizates of TUN219-2C1-hFc were stored at RT or - 80°C for 30 days and afterwards compared with the fresh antibody in SEC or indirect ELISA. Data derived relative affinity values (EC_50_) and monomer species percentages are shown in **Supplementary Table 2**.

Storage of TUN219-2C1-hFc in 1 x PBS solution at RT resulted in a drop of the monomer species fraction measured in SEC from 72.8 ± 0.7% to 13.3 ± 0.5%, mostly due to the increased formation of degradation products. These data correlate with the impaired binding activity measured in antigen ELISA (**Figure 2A**). By comparing the EC_50_ value with the one of fresh antibody sample, a 3.4-fold decrease in activity could be determined (**Supplementary Table 2**). In contrast, lyophilizing TUN219-2C1-hFc in 0.01 M sodium phosphate buffer with a pH of 7.4 or 7.8 and 2% trehalose, as well as 0.1 M sodium phosphate buffer with a pH of 7.8 and no excipient, preserved unaltered the original binding activity after 30 days at RT (**Supplementary Table 2**).

In line with their antibody binding activity, the same samples did not show any formation of degradation products, however, if no sugar was present in the formulation and sodium phosphate concentration was raised to 0.1 M, a significant increase in HMMS was observed after thirty days at RT (**Figure 2B**), reducing the monomer content to only 50.0 ± 0.5%. The corresponding samples stored at - 80°C showed only in one of three cases a slightly higher amount of HMMS (**Figure 2B**). For both formulations containing 2% trehalose, no significant differences in the MM distribution could be detected compared to the control sample.

Due to the lower inter-replicate variation in the binding activity measured in ELISA for TUN219-2C1-hFc lyophilized in 0.01 M sodium phosphate buffer pH 7.4 plus 2% trehalose, this formulation was preferred to the same at pH 7.8 for further experiments. These results demonstrate that shelf-life of the TUN219-2C1-hFc as model antibody at room temperature could be significantly increased by the new freeze-drying protocol. The protocol was successfully adopted to lyophilize protein in 2 mL glass vials with a diameter of 11 mm where the same degree of preservation of the original protein MM distribution and binding activity and identical cake appearance was obtained (**Supplementary Figure 1**).

### Protocol Validation With Other scFv-Fc Antibodies

**Figure 3:**
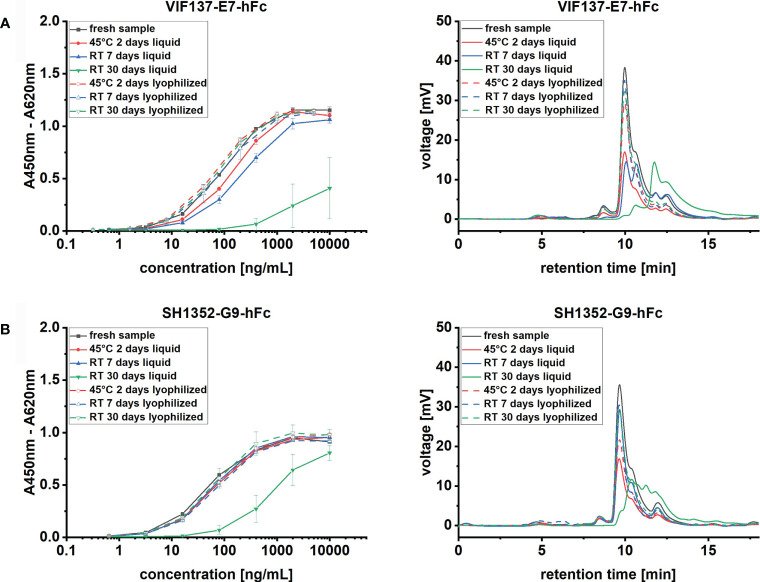
Time course analysis of different scFv-hFc mAbs activity and MM distribution upon storage test. Antibody proteins at 0.5 mg/mL concentration were stored at RT or 45°C, respectively for 7 - 30 or 2 days, in liquid (solid lines) or solid, freeze-dried (dashed lines), state. Left panel: mAb titration binding curves on 100 ng immobilized antigen, right panel, representative SEC chromatograms for each condition for respectively scFv-Fc mAbs **(A)** VIF137-E7-hFc and **(B)** SH1352-G9-hFc. All samples after storage are compared to fresh material (black line). ELISA curve error bars result from the analysis of 3 independently lyophilized or liquid stored samples.

The final lyophilization protocol was then applied to two other scFv-hFcs, phage display-derived antibodies VIF137-E7-hFc (Fühner et al., 2018) and SH1352-G9-hFc (Russo et al., 2018) against *Clostridioides difficile* toxin B and zebrafish cadherin 2, respectively. Stability profiles of lyophilized and liquid samples were evaluated after 7 and 30 days at RT or 2 days at 45°C as accelerated long-term stability test (**Figure 3**).

Like with TUN219-2C1-hFc, lyophilization of VIF137-E7-hFc and SH1352-G9-hFc resulted in adequate cakes, which did not alter their appearance over 30 days at RT (data not shown). When VIF137-E7-hFc was stored dissolved in 1 x PBS at RT increased degradation already occurred within the first 7 days. At this point, less than two thirds of the initial monomer content was still present, which consequently resulted in reduced binding in ELISA as well (**Figure 3A**). After 30 days, this effect was even more pronounced. Residual monomer content dropped to as little as 2.1 ± 1.6% (**Supplementary Table 3**), whereas most of the detected species were degradation products (**Figure 3A**). Thus, the corresponding antibody binding activity was almost completely abolished. SH1352-G9-hFc showed comparable behavior, albeit the antibody appeared to be stable for one week at RT (**Figure 3B**). After 30 days, monomers were drastically reduced compared to the initial value (from 61.4 ± 1.0% to 1.6 ± 0.4%) and the EC_50_ was found to be 757 ng/mL compared to 56 ng/mL of the fresh antibody (**Supplementary Table 3**). Moreover, although VIF137-E7-hFc showed reduced binding with respect to the accelerated long-term test, prolonged storage at RT seemed to have a stronger impact on the stability of both antibodies than a shorter incubation at higher temperature (45°C). In contrast to the liquid storage form, shelf-life was extended for both scFv-hFc antibodies by means of lyophilization. Here, initial binding properties have been preserved and no significantly increased amounts of HMMS and low molecular mass species (LMMS) were observed in the SEC chromatogram.

### ScFv-Fc and IgG Format Comparison

**Figure 4:**
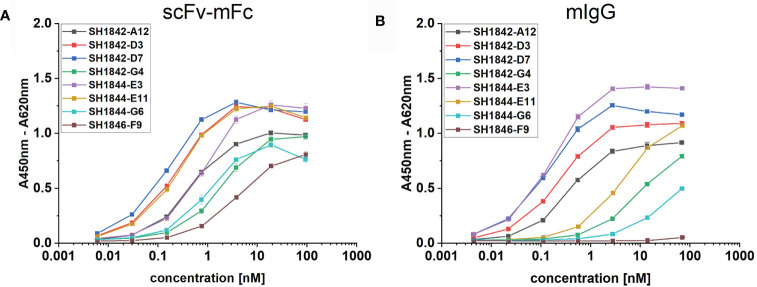
Comparison of scFv-Fc or IgG format in ELISA binding assay. Eight mAbs were produced respectively as **(A)** scFv-mFc (mIgG2a-Fc) or **(B)** full mIgG2a. 500 pg/mL to 10 µg/mL were incubated against 100 ng immobilized hFc antigen, representing molar concentrations spanning from **(A)** 5.95 – 6.08 pM to 92.92 – 95.04 nM and **(B)** 4.49 – 4.36 pM to 68.13 – 70.13 nM due to slight differences in molecular masses.

In order to compare format-dependent binding activity, we converted eight anti-human IgG scFv antibodies from various gene families (**Table 3**) into scFv-mouseFc or mouseIgG formats. All these clones, made exception for SH1844-E3 (anti-human IgG1), were chosen due to previous observations of poor stability (data not shown). Conversion into the IgG format impaired the binding activity of half of the studied antibody clones (SH1842-G4, SH1844-E11, SH1844-G6 and SH1846-F9), as represented by the highly increased EC_50_ values or even complete loss of activity in ELISA compared to the corresponding scFv-Fcs (**Figure 4**). In contrast, SH1844-E3 showed a 4.6 fold decrease in EC_50_ upon format change (**Table 3**).

**Table 3:** Phage-display derived anti-hIgG antibodies used for format comparison.

Antibody clone	V-VH	D-VH	J-VH	V-VL	J-VL	Yield [mg/L]		EC_50_ [nM]	
						scFv-mFc	mIgG	scFv-mFc	mIgG
**SH1842-A12**	IGHV1-18*01	IGHD3-3*01	IGHJ4*02	IGLV2-11*01	IGLJ3*01	140.0	15.2	0.46	0.35
**SH1842-D3**	IGHV3-9*01	IGHD2-2*02	IGHJ4*02	IGLV7-43*01	IGLJ3*02	152.8	58.8	0.25	0.23
**SH1842-D7**	IGHV3-30*18	IGHD3-16*01	IGHJ4*02	IGLV1-40*01	IGLJ3*01	108.0	24.8	0.12	0.11
**SH1842-G4**	IGHV3-30*18	IGHD3-16*01	IGHJ4*02	IGLV3-21*03	IGLJ3*01	143.2	15.6	1.90	n.s.b.
**SH1844-E3**	IGHV3-7*01	IGHD4-23*01	IGHJ3*02	IGKV1-NL1*01	IGKJ1*01	148.0	22.8	0.73	0.16
**SH1844-E11**	IGHV3-23*04	IGHD1-26*01	IGHJ4*02	IGKV3D-11*01	IGLKJ3*01	123.2	40.8	0.20	4.39
**SH1844-G6**	IGHV3-33*01	IGHD2-21*01	IGHJ4*02	IGKV3-20*01	IGKJ5*01	84.0	72.0	0.82	n.s.b.
**SH1846-F9**	IGHV1-46*03	IGHD6-13*01	IGHJ4*02	IGKV1D-39*01	IGKJ4*01	129.6	35.6	2.31	n.b.

Antibodies from various gene families were produced as mIgG2a-Fc fusion or full mIgG2a in Expi293F suspension cells. Yield values are extrapolated from 50 mL scale. “n.s.b.” indicates non-sigmoidal weak binding. No binding is abbreviated as “n.b.”. The asterisk is typically used for indicating the allele of an antibody gene.

**Figure 5:**
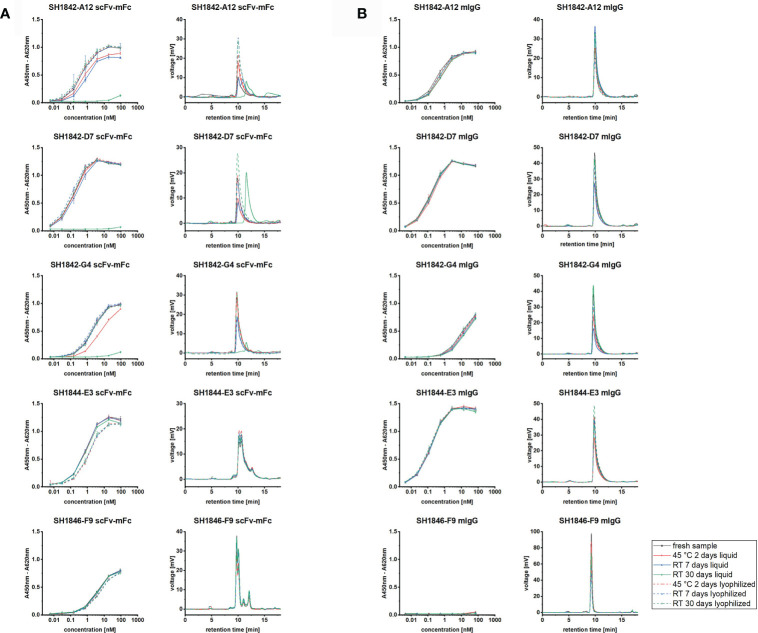
Storage stability of scFv-Fc or IgG antibodies. Upon storage at RT or 45°C, respectively for 7-30 or 2 days, in liquid (solid lines) or freeze-dried (dashed lines) state, five antibody clones were studied for their residual binding activity in ELISA and MM distribution in SE-HPLC in **(A)** scFv-mFc2a format or **(B)** full mIgG2a format. For SE-HPLC are shown representative chromatograms for each condition. ELISA curve error bars result from the analysis of 3 independently lyophilized or liquid stored samples.

Shelf-life under different storage conditions was assessed for SH1842-A12, SH1842-D7, SH1842-G4, SH1844-E3 and SH1846-F9. The format change led to an increase in the monomer fraction after storage at RT for 30 days independent of the storage form (**Figure 5**). Moreover, none of the IgG samples incubated two days at 45°C was affected by this treatment. On the other hand, the MM distribution assessment revealed that three out of five antibody clones in the scFv-mFc format presented clear signs of degradation after storage at RT for 30 days, including SH1842-G4, one of the clones which cannot be converted into IgG. The known relation between increase of the LMMS fraction and reduction of the binding activity found confirmation also in this case. At the same time, the inverse relation could not be always established, meaning that an antibody that did not show any difference in its MM distribution after storage may show reduced binding. This was the case with SH1842-G4 that lost binding activity due to exposure to high temperatures (45°C) for two days while not showing any change in the monomer fraction, aggregation or degradation. In contrast, by means of lyophilization the shelf-life of all unstable scFv-mFc was prolonged. Here, after the whole period of storage, the EC_50_ as well as monomer portion were still similar compared to the fresh samples. All stable antibodies, such as IgGs, were not intrinsically harmed by the lyophilization process.

**Figure 6:**
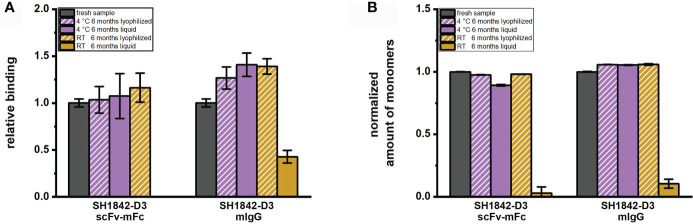
Six months storage impact on mAb activity and molecular species composition. **(A)** Bar chart representing binding measured in titration ELISA on 100 ng immobilized antigen. EC_50_ values as quantification of the relative antibody affinity were reciprocally normalized to the reference material (fresh sample). **(B)** In the bar chart is shown the monomer percentage per each antibody stored in different conditions as indirect measure of aggregation or degradation occurred over time and normalized to the monomer percentage of the fresh sample. Error bars result from the analysis of 2 to 3 independently lyophilized or liquid stored samples.

In addition, an extended storage test for 6 months was carried out for clone SH1842-D3 in scFv-mFc as well as mIgG format. The antibody’s binding (**Figure 6A**) was strongly diminished (mIgG) or completely abolished (scFv-mFc) upon storage at RT in solution. SH1842-D3 mIgG antibody degraded over time when stored at RT in solution (**Figure 6B**). In contrast to RT, liquid storage at 4°C did not impair the molecules’ EC_50,_ whereas the monomer percentage was slightly reduced for the scFv-mFc format. For both formats, lyophilization was also successful in preserving the binding activity and amount of monomers of SH1842-D3 at 4°C and RT to the levels of the fresh sample.

## Discussion

In this study, we describe the development of a new lyophilization protocol to extend the shelf-life of antibodies in the scFv-Fc format. Furthermore, we show that for specific clones the format conversion into the more stable IgG format impairs or completely abolishes binding, making lyophilization the method of choice to increase these clones’ shelf life.

Despite eventually extending shelf-life of proteins, a freeze-drying process intrinsically contains numerous protein destabilizing factors. These effects are minimized by creating a tailor-made protective environment for the protein of interest. A total of 55 different buffer formulations were screened to systematically determine the optimal scFv-Fc conditions for freeze-drying and successful reconstitution. Per each formulation was included maximum one stabilizing excipient and the concentration of model scFv-Fc protein was kept at 0.5 mg/mL. The model antibody (TUN219-2C1-hFc) was selected as candidate as we observed that liquid storage at RT for 30 days in 1 x PBS significantly impairs its binding activity in ELISA as result of a reduction in the antibody monomer fraction (**Figure 2A**). For comparability and effectiveness, conservative lyophilization cycles for freeze-drying in MTPs or glass vials were adopted along the whole study, with adjustments dependent on the lyophilization machine and were based on performing the sublimation step well below the excipients’ predicted glass transition temperatures (Bjelošević et al., 2020).

Cake collapse was adopted as first exclusion criterion. Collapse does not ultimately result in loss of protein activity, but is related to adverse elevated residual moisture and prolonged reconstitution times. In addition, a survey among participants with varying expertise in the field of lyophilization, carried out by Patel and colleagues (Patel et al., 2017), found major collapse to be a common cause for product rejection.

Only 24/55 formulations resulted in the formation of structurally defined and well reconstitutable lyophilizates (**Figure 1** and **Table 1**). While sodium phosphate, especially in the presence of sugar excipients, resulted in robust formation of cakes, formulations based on 1 x PBS, Tris-HCl or 0.1 M potassium phosphate buffers led indifferently to collapse of the cake structure. Similarly to our findings, Sarciaux and colleagues described the deep differences in cake formation depending on the use of different phosphate buffers (Sarciaux et al., 1999), occurring even if primary drying is carried out at lower temperatures.

Adequate lyophilizates were then analyzed by SEC and antigen ELISA. In this study, aggregation behavior of TUN219-2C1-hFc during freeze-drying was overall slightly superior using sodium phosphate over 0.01 M potassium phosphate, 1 x PBS and Tris-HCl and was therefore selected as final buffer system. In contrast, (Sarciaux et al., 1999) found potassium phosphate to be more suitable than sodium phosphate in aggregation behavior of bovine IgG during freeze-drying. They further describe decreased stability, in case phosphate buffers were used in combination with tonicity agents like KCl and NaCl. This suggests avoidance of PBS as lyophilization buffer for antibodies. However, we could detect only minor decreased monomer contents for a 1 x PBS formulation.

PH changes upon selective crystallization of phosphate buffer components during freezing have been discussed for more than half a century (van den Berg and Rose, 1959). However, in the last decades this topic has drawn increased attention as sodium phosphate buffers are broadly used in formulations for freeze-drying therapeutic proteins (Schwegman et al., 2005; Mensink et al., 2017), although freezing-induced drops of 3 to 3.5 pH units have been reported (Sarciaux et al., 1999; Pikal-Cleland and Carpenter, 2001; Kolhe et al., 2010). The impact of pH shifts on protein stability remains arguable. Freeze-thaw and lyophilization studies using model enzymes (Jiang and Nail, 1998; Pikal-Cleland et al., 2000; Thorat et al., 2020) or BSA (Thorat et al., 2020) showed decreased retention of stable and active proteins upon freezing in sodium phosphate buffers, which provides compelling reasons for avoidance of sodium phosphate in protein formulations for freeze-drying.

However, one has to particularly consider the protein of interest’s nature as well as the composition of a formulation and process parameters. 0.01 M sodium phosphate was found to influence the pH during freezing less drastically than the equivalent 0.1 M buffer (Bhatnagar et al., 2007). In addition, it is known that polymers and sugars like sucrose (Anchordoquy and Carpenter, 1996) or trehalose (Thorat and Suryanarayanan, 2019) can prevent mentioned pH shifts by inhibiting selective crystallization of phosphate buffer components. Rapid freezing techniques are furthermore favored in order to reduce exposure time of proteins towards shifted pH values (Pikal-Cleland et al., 2000). Although potassium phosphate and Tris-HCl buffers do not result in drops of pH values upon freezing, solutions become more alkaline (Bhatnagar et al., 2007; Kolhe et al., 2010), which might be a bigger threat for integrity of TUN219-2C1 than low pH, due to the risk of formation of nonreducible cross-linking at basic pH (Wang et al., 2007).

Surprisingly, we found that 0.1 M sodium phosphate pH 7.8 without any cryoprotectant still adequately retained binding and MM distribution of TUN219-2C1 after freeze-drying, although the composition of this particular formulation is prone to cause acidification during freezing. This indicates that possible pH shifts play a minor role in threatening stability of this scFv-Fc antibody or aggregation was reversible upon reconstitution. Likewise, (Sarciaux et al., 1999) found that aggregation of bovine IgG as result of lyophilization was not mediated by sodium phosphate induced acidification. It is known, that aggregation of monoclonal antibodies can occur at acidic conditions (Singla et al., 2016). However, first signs of denaturation of hIgG1-Fc CH_2_ were observed only at pH 3.5 and below by (Latypov et al., 2012) using NMR. Thereof, it is likely that at least for TUN219-2C1-hFc, destabilization only happens well below the lower limit of a predicted pH shift. Still, this assumption cannot be universally applied to other scFv-Fc molecules, i. e. hIgG2-Fc was found to be less stable than hIgG1-Fc (Latypov et al., 2012). Moreover, the variable regions of an scFv-Fc do contribute to antibody stability as well (Manning et al., 2010). Hence, addition of polymers or sugars should be considered in particular for antibodies with unknown pH stability.

The benefit of sugar-containing formulations was evident after lyophilizates were stored at RT for 30 days. In contrast to sugar-containing formulations, the absence of a stabilizer diminished the amount of monomers of TUN219-2C1-hFc by almost a third (from 72.5 to 50 %) during storage (**Supplementary Table 2**). Here, a large portion of the scFv-Fc antibody was aggregated (**Figure 2B**). ELISA data did not reveal any loss in binding activity for aggregated material (**Figure 2B**), highlighting the need of complementing binding assays with the study of the physical state of the molecule. Indeed, aggregation may still affect the long term stability of the molecule and, most importantly, it may constitute a source of unwanted immunogenicity (Xu et al., 2019). For proteins, removal of water is a double-edged sword. While hydrolytic reactions are decelerated, the native conformation has to be stabilized by hydrogen bonds. Here, the hydroxyl groups of lyoprotectants like trehalose and sucrose can serve as water replacement (Harris et al., 2004; Emami et al., 2018). In addition, amorphous excipients embed the protein of interest in a highly viscous glassy matrix, kinetically hindering any unfolding or other degradation processes (Bhatnagar et al., 2007). In line with major findings in the fields, our results emphasize the need for a stabilizing excipient in the solid state to prevent aggregation during storage.

Within the two sugars excipients tested, trehalose was preferred over sucrose, as collapse of sucrose containing lyophilizates was observed after 30 days at RT. In contrast, using trehalose as excipient resulted in unchanged cake appearance over time. Because water acts as plasticizer, T_g_ of formulations decreases in accordance to their moisture content (Skrabanja et al., 1994; Patel et al., 2017). Sucrose has not only a much lower T_g_ (Bjelošević et al., 2020), but is also more hygroscopic than trehalose (Ryu et al., 2007). Thereof, both high amounts of residual moisture after lyophilization as well as diffusion of moisture through rubber stoppers into the vial (Vromans and van Laarhoven, 1992) can be considered as origin of the observed collapse in sucrose based formulations.

Finally, we found 0.01 M sodium phosphate with the addition of 2% trehalose and an antibody concentration of 0.5 mg/mL to be the most protective formulation for freeze-drying TUN219-2C1-hFc. Lyophilization using this formulation was successful in both MTP stripes as well as different glass vials.

Since our screening approach impeded mutual cycle development, process optimization was not performed within the scope of this study. However, freeze-drying is an expensive and time-consuming process that aims for optimal process parameters. Primary drying is usually the longest step of freeze-drying. Here, an only 1 °C increase of product temperature can reduce the duration for this stage by 13 % (Depaz et al., 2016). For this reason, drying is recommended to be performed at temperatures as high as possible without risking protein stability. This is usually slightly below T_c_ (Tang and Pikal, 2004). Thus, in further studies, temperatures for glass transition or collapse of the selected formulation should be measured. In addition, duration of primary drying can be further shortened by sublimation end point determination (Daoussi et al., 2009). Finally, another critical parameter for shelf-life in the dried state is residual moisture (Jiang and Nail, 1998). In order to assess stability of the lyophilizate during storage, in particular at elevated temperatures, determination of residual moisture content after secondary drying would be of interest.

The protocol’s broad applicability to other scFv-Fc molecules was successfully validated with VIF137-E7-hFc and SH1352-G9-hFc, two recombinant human antibodies against *Clostridioides difficile* toxin B and zebrafish cadherin 2, respectively. Liquid storage exhibited limited shelf-life for both antibodies, outlined by degradation (**Figure 3**) and consequently impaired binding in ELISA (**Figure 3**). These findings further state the urge for stabilization of scFv-Fc antibodies. Here, our novel lyophilization protocol perfectly preserved the antibodies’ original properties for at least one month and at elevated temperatures (45°C) for two days (**Figure 3**).

In order to assess format-dependent binding activity, we selected a panel of eight antibody clones (**Table 3**) produced as scFv-Fc (mouse IgG2a-Fc) as well as full mouse IgG2a. For a more consistent analysis of the binding properties, we selected antibodies specific to the same target, human IgG, but diverse in their VH and VL gene families. Six of these clones were then compared in their long-term stability in the freeze-dried or soluble state for up to one month (**Figure 5**) or half a year (**Figure 6**), to verify the lyophilization protocol’s applicability to different antibody molecules.

In average, the scFv-Fc or IgG antibodies expressed in mammalian cells under the control of the same promoter, yielded 125.1 mg per liter of culture and 35.7 mg, respectively. Even the best produced IgG did not yield as much as the worst scFv-Fc producer. In transient expression systems the bottleneck in production is independent from mRNA levels and relies on the complexity of translational and post-translational modifications of a certain molecule (Mason et al., 2012). In our case, the heavy and light chain pairing plus the higher number of disulfide bonds present in IgG molecules probably were responsible for the lower yields. Despite targeted mutagenesis (Mason et al., 2012) or modifications in the folding machinery in the secretory pathway are possible to improve IgG yields. However, they are clone-specific and laborious (Kaneyoshi et al., 2019), so they cannot substitute the advantages of scFv-Fc if rapid small scale production of bivalent Fc-carrying antibodies is needed in high throughput settings.

During recombinant naïve antibody gene library construction, VH and VL genes from donor B-cells are often shuffled, increasing original antibody diversity, but also causing the loss of original chains pairing (Lerner, 2016). Hence, antibodies obtained from shuffled scFv libraries may not recapitulate the donor’s naturally paired VH/VL repertoire. However, correct pairing of VH and VL can be crucial for antigen recognition (Goldstein et al., 2019). Moreover, randomly paired scFvs were found more likely to have inferior developability properties upon conversion into full-length IgGs (Adler et al., 2018). In the scFv molecule, VH and VL are connected by a flexible linker, but oriented according to a combination-specific angle (Abhinandan and Martin, 2010). Consequently, while antibody selection results in the identification of functional scFv molecules, it is not known if in a certain chain combination, the VH and VL are reciprocally oriented as in the respective full IgG antibody they originated from. This implies that by converting an scFv into IgG, we may change the packing angle between VH and VL, potentially improving or harnessing the binding. By comparing the binding activity in ELISA of each scFv-Fc to the corresponding IgG (**Supplementary Table 3** and **Figure 4**), we observed that 4/8 antibodies showed reduced if not completely abolished binding (SH1846-F9). It is possible to speculate that for those four antibodies VH/VL orientation differs from the one in IgG format as previously described (Menzel et al., 2008; Steinwand et al., 2014; Bertoglio et al., 2021). Only in 1/8 cases (SH1844-E3) we detected an increase in the apparent binding affinity (4.6 folds decrease in EC_50_). As before, this could be related to a possible change in the VH/VL orientation, this time beneficial. Nevertheless, our data support at least one other conclusion. In the absence of storage at RT for several days or weeks, SH1844-E3-Fc is mostly presented as a mixture of two defined molecular species: the monomer and a smaller proteolytic product (**Figure 5**), which could be less functional (i. e. loss of one scFv moiety), or not detectable by secondary antibodies (i. e. loss of Fc-part). On the contrary, the corresponding IgG is composed by a single monomeric species, which in equimolar concentration would result in increased binding even in the absence of an affinity improvement. Further studies aimed at measuring the affinity of monomeric SH1844-E3 scFv or Fab should be aimed at investigating this possibility.

When comparing antibody protein MM distribution of SH1842-A12, SH1842-D7, SH1842-G4, SH1844-E3 and SH1846-F9 before and after lyophilization, we show that neither the buffer exchange nor the freeze-drying process and storage over a period of 30 days at RT or 2 days at 45°C neither harmed the IgG nor the scFv-Fc molecules (**Figure 5**). As previously shown for human IgG1-Fc fused scFvs, lyophilization preserved the original physical state and binding activity over the complete storage duration also for the 3/5 scFv-Fc (mIgG2a-Fc) molecules that showed almost complete degradation when stored in solution (**Figure 5**). To observe also a reduction in the binding activity of IgG, we had to wait 6 months, where SH1842-D3 stored in solution at RT showed a 9.4 times reduction in the monomer fraction and impaired antigen recognition in ELISA (**Figure 6**). The equivalent lyophilized molecule, again, showed unaltered properties compared to the corresponding material stored at - 80°C. The higher overall stability of IgGs over scFv-Fcs is in line with the intrinsic nature of the latter, which lacks the stabilizing CH1-CL interface and interchain connecting disulfide bond (Röthlisberger et al., 2005). Interestingly, all four lambda, but not the two kappa Abs in the scFv-Fc format degraded over storage duration at RT when stored in solution. Also, 3/4 lambda antibodies preserved their binding activity during format conversion from scFv-Fc into IgG, while 3/4 kappa Abs lost mostly or completely their binding. These findings should be further investigated testing a larger set of antibodies from different naïve antibody libraries.

Overall, while IgG conversion improved the antibodies’ stability and increased their shelf-life (**Figure 5**), it also resulted in a drastic reduction of the binding activity for 4/8 studied antibodies. If we combine this knowledge with the fact that in modern medicine the use of scFv-based, instead of Fab-based, therapeutic molecules are increasing (Brinkmann and Kontermann, 2017; Guedan et al., 2019) it becomes evident the need for a lyophilization protocol to expand scFv-Fc shelf-life. Additionally, lyophilized scFv-Fc products with high stability at RT and up to 45°C would simplify storage and allow shipments in the absence of refrigeration chain, therefore, facilitating the use of this material in countries where equipped laboratories are not densely distributed on the territory. Shipping in the absence of dry-ice is also a cost benefit and more sustainable.

## Data Availability Statement

The raw data supporting the conclusions of this article will be made available by the authors, without undue reservation.

## Author Contributions

K-TS, GR, and SD conceptualized the study. K-TS, TK, DM, SP, and MB performed and designed experiments. K-TS, TK, EW, and GR analyzed data. J-HG, PM, and MH advised on experimental design and data analysis. K-TS, GR, and SD wrote the manuscript. All authors contributed to the article and approved the submitted version.

## Conflict of Interest

GR, EW, and SD are co-founders and shareholders of Abcalis GmbH. SD and MH are co-founders and shareholders of mAb-factory GmbH, YUMAB GmbH and NordenVaccines GmbH. DM is co-founder and shareholder of mAb-factory GmbH.

The remaining authors declare that the research was conducted in the absence of any commercial or financial relationships that could be construed as a potential conflict of interest.

## Publisher’s Note

All claims expressed in this article are solely those of the authors and do not necessarily represent those of their affiliated organizations, or those of the publisher, the editors and the reviewers. Any product that may be evaluated in this article, or claim that may be made by its manufacturer, is not guaranteed or endorsed by the publisher.

## Supplementary Material

Supplementary Figure 1 Different glass vials size lyophilization comparison test. **(A)** Cake appearance immediately post lyophilization or after 30 days RT storage of 315 µL 0.5 mg/mL TUN219-2C1-hFc Ab solution in a cylindrical glass vial having a base-diameter of 15 mm and **(C)** 189 µL of the same material in a glass vial with 11 mm base-diameter. **(B)** Titration ELISA and SEC or SE-HPLC chromatogram of Ab lyophilized in 15 mm vials or **(D)** 11 mm vials, respectively. Each lyophilized Ab is compared, after reconstitution, to fresh material.

Supplementary Table 1 Summary of EC_50_ values and percentages of monomers after lyophilization of TUN219-2C1-hFc in MTPs using different formulations. Values for lyophilizates are the mean of 3 individually lyophilized samples. Values of formulations that were either excluded by previous selection criteria or have not been in the scope of this study were not determined (“n.d.”).

Supplementary Table 2 Summary of EC_50_ values and percentages of monomers after lyophilization of TUN219-2C1-hFc in 2 mL (15 mm) glass vials using different formulations and subsequent storage for 30 days. Values for lyophilizates are the mean of 3 individually lyophilized samples. Values of formulations that were either excluded by previous selection criteria or have not been in the scope of this study were not determined (“n.d.”).

Supplementary Table 3 Summary of EC_50_ values and percentages of monomers after storage of VIF137-E7-hFc and SH1352-G9-hFc. “n.s.b.” refers to non-sigmoidal binding curves.

## References

[B1] Abhinandan K. R. Martin A. C. R. (2010). Analysis and Prediction of VH/VL Packing in Antibodies. Protein Eng. Design. Selection. PEDS. 23 (9), 689–697. doi: 10.1093/protein/gzq043 20591902

[B2] Adler A. S. Bedinger D. Adams M. S. Asensio M. A. Edgar R. C. Leong R.. (2018). A Natively Paired Antibody Library Yields Drug Leads With Higher Sensitivity and Specificity Than a Randomly Paired Antibody Library. mAbs 10 (3), 431–443. doi: 10.1080/19420862.2018.1426422 29376776 PMC5916548

[B3] Alfaleh M. A. Alsaab H. O. Mahmoud A. B. Alkayyal A. A. Jones M. L. Mahler S. M.. (2020). Phage Display Derived Monoclonal Antibodies: From Bench to Bedside. Front. Immunol. 11, 1986. doi: 10.3389/fimmu.2020.01986 32983137 PMC7485114

[B4] Anchordoquy T. J. Carpenter J. F. (1996). Polymers Protect Lactate Dehydrogenase During Freeze-Drying by Inhibiting Dissociation in the Frozen State. Arch. Biochem. Biophys. 332 (2), 231–238. doi: 10.1006/abbi.1996.0337 8806730

[B5] Arndt K. M. Müller K. M. Plückthun A. (1998). Factors Influencing the Dimer to Monomer Transition of an Antibody Single-Chain Fv Fragment. Biochemistry 37 (37), 12918–12926. doi: 10.1021/bi9810407 9737871

[B6] Austerberry J. I. Thistlethwaite A. Fisher K. Golovanov A. P. Pluen A. Esfandiary R.. (2019). Arginine to Lysine Mutations Increase the Aggregation Stability of a Single-Chain Variable Fragment through Unfolded-State Interactions. Biochemistry 58 (32), 3413–3421. doi: 10.1021/acs.biochem.9b00367 31314511

[B7] Barbas C. F. Kang A. S. Lerner R. A. Benkovic S. J. (1991). Assembly of Combinatorial Antibody Libraries on Phage Surfaces: The Gene III Site. Proc. Natl. Acad. Sci. U.S.A. 88 (18), 7978–7982. doi: 10.1073/pnas.88.18.7978 1896445 PMC52428

[B8] Bernhard W. El-Sayed A. Barreto K. Gonzalez C. Fonge H. Geyer C. R. (2019). Near Infrared Imaging of Epidermal Growth Factor Receptor Positive Xenografts in Mice With Domain I/II Specific Antibody Fragments. Theranostics 9 (4), 974–985. doi: 10.7150/thno.30835 30867810 PMC6401412

[B9] Bertoglio F. Meier D. Langreder N. Steinke S. Rand U. Simonelli L.. (2021). SARS-CoV-2 Neutralizing Human Recombinant Antibodies Selected From Pre-Pandemic Healthy Donors Binding at RBD-ACE2 Interface. Nat. Commun. 12 (1), 1577. doi: 10.1038/s41467-021-21609-2 33707427 PMC7952403

[B10] Bhatnagar B. S. Bogner R. H. Pikal M. J. (2007). Protein Stability During Freezing: Separation of Stresses and Mechanisms of Protein Stabilization. Pharm. Dev. Technol. 12 (5), 505–523. doi: 10.1080/10837450701481157 17963151

[B11] Bjelošević M. Zvonar Pobirk A. Planinšek O. Ahlin Grabnar P. (2020). Excipients in Freeze-Dried Biopharmaceuticals: Contributions Toward Formulation Stability and Lyophilisation Cycle Optimisation. Int. J. Pharmaceut. 576, 119029. doi: 10.1016/j.ijpharm.2020.119029 31953087

[B12] Borrebaeck C. A. K. (2000). Antibodies in Diagnostics – From Immunoassays to Protein Chips. Immunol. Today 21 (8), 379–382. doi: 10.1016/S0167-5699(00)01683-2 10916140

[B13] Breitling F. Dübel S. Seehaus T. Klewinghaus I. Little M. (1991). A Surface Expression Vector for Antibody Screening. Gene 104 (2), 147–153. doi: 10.1016/0378-1119(91)90244-6 1916287

[B14] Brinkmann U. Kontermann R. E. (2017). The Making of Bispecific Antibodies. mAbs 9 (2), 182–212. doi: 10.1080/19420862.2016.1268307 28071970 PMC5297537

[B15] Brinkmann U. Reiter Y. Jung S. H. Lee B. Pastan I. (1993). A Recombinant Immunotoxin Containing a Disulfide-Stabilized Fv Fragment. Proc. Natl. Acad. Sci. U.S.A. 90 (16), 7538–7542. doi: 10.1073/pnas.90.16.7538 8356052 PMC47177

[B16] Cai Y. Zhang J. Lao X. Jiang H. Yu Y. Deng Y.. (2016). Construction of a Disulfide-Stabilized Diabody Against Fibroblast Growth Factor-2 and the Inhibition Activity in Targeting Breast Cancer. Cancer Sci. 107 (8), 1141–1150. doi: 10.1111/cas.12981 27251178 PMC4982589

[B17] Colandene J. D. Maldonado L. M. Creagh A. T. Vrettos J. S. Goad K. G. Spitznagel T. M. (2007). Lyophilization Cycle Development for a High-Concentration Monoclonal Antibody Formulation Lacking a Crystalline Bulking Agent. In J. Pharm. Sci. 96 (6), 1598–1608. doi: 10.1002/jps.20812 17117409

[B18] Cordoba A. J. Shyong B.-J. Breen D. Harris R. J. (2005). Non-Enzymatic Hinge Region Fragmentation of Antibodies in Solution. J. Chromatograp. B Anal. Technol. Biomed. Life Sci. 818 (2), 115–121. doi: 10.1016/j.jchromb.2004.12.033 15734150

[B19] Crowe L. M. Reid D. S. Crowe J. H. (1996). Is Trehalose Special for Preserving Dry Biomaterials? Biophys. J. 71 (4), 2087–2093. doi: 10.1016/S0006-3495(96)79407-9 8889183 PMC1233675

[B20] Daoussi R. Vessot S. Andrieu J. Monnier O. (2009). Sublimation Kinetics and Sublimation End-Point Times During Freeze-Drying of Pharmaceutical Active Principle With Organic Co-Solvent Formulations. Chem. Eng. Res. Design. 87 (7), 899–907. doi: 10.1016/j.cherd.2008.09.007

[B21] Depaz R. A. Pansare S. Patel S. M. (2016). Freeze-Drying Above the Glass Transition Temperature in Amorphous Protein Formulations While Maintaining Product Quality and Improving Process Efficiency. J. Pharm. Sci. 105 (1), 40–49. doi: 10.1002/jps.24705 26580140

[B22] Ecker D. M. Jones S. D. Levine H. L. (2015). The Therapeutic Monoclonal Antibody Market. mAbs 7 (1), 9–14. doi: 10.4161/19420862.2015.989042 25529996 PMC4622599

[B23] Emami F. Vatanara A. Park E. J. Na D. H. (2018). Drying Technologies for the Stability and Bioavailability of Biopharmaceuticals. Pharmaceutics 10 (3), 1–22. doi: 10.3390/pharmaceutics10030131 PMC6161129 30126135

[B24] Engler C. Kandzia R. Marillonnet S. (2008). A One Pot, One Step, Precision Cloning Method With High Throughput Capability. PloS One 3 (11), e3647. doi: 10.1371/journal.pone.0003647 18985154 PMC2574415

[B25] Franks F. (1998). Freeze-Drying of Bioproducts: Putting Principles Into Practice. Eur. J. Pharmaceut. Biopharmaceut. 45 (3), 221–229. doi: 10.1016/S0939-6411(98)00004-6 9653626

[B26] Frenzel A. Hust M. Schirrmann T. (2013). Expression of Recombinant Antibodies. Front. Immunol. 4, 217. doi: 10.3389/fimmu.2013.00217 23908655 PMC3725456

[B27] Fühner V. Heine P. A. Helmsing S. Goy S. Heidepriem J. Loeffler F. F.. (2018). Development of Neutralizing and Non-Neutralizing Antibodies Targeting Known and Novel Epitopes of TcdB of Clostridioides Difficile. Front. Microbiol. 9, 2908. doi: 10.3389/fmicb.2018.02908 30574127 PMC6291526

[B28] Ghose S. Hubbard B. Cramer S. M. (2007). Binding Capacity Differences for Antibodies and Fc-Fusion Proteins on Protein A Chromatographic Materials. Biotechnol. Bioeng. 96 (4), 768–779. doi: 10.1002/bit.21044 16817242

[B29] Goldstein L. D. Chen Y.-J. J. Wu J. Chaudhuri S. Hsiao Y.-C. Schneider K.. (2019). Massively Parallel Single-Cell B-Cell Receptor Sequencing Enables Rapid Discovery of Diverse Antigen-Reactive Antibodies. Commun. Biol. 2, 304. doi: 10.1038/s42003-019-0551-y 31428692 PMC6689056

[B30] Grilo A. L. Mantalaris A. (2019). The Increasingly Human and Profitable Monoclonal Antibody Market. Trends Biotechnol. 37 (1), 9–16. doi: 10.1016/j.tibtech.2018.05.014 29945725

[B31] Guedan S. Calderon H. Posey A. D. Maus M. V. (2019). Engineering and Design of Chimeric Antigen Receptors. Mol. Ther. Methods Clin. Dev. 12, 145–156. doi: 10.1016/j.omtm.2018.12.009 30666307 PMC6330382

[B32] Harris R. J. Shire S. J. Winter C. (2004). Commercial Manufacturing Scale Formulation and Analytical Characterization of Therapeutic Recombinant Antibodies. Drug Dev. Res. 61 (3), 137–154. doi: 10.1002/ddr.10344

[B33] Hay C. E. Ewing L. E. Hambuchen M. D. Zintner S. M. Small J. C. Bolden C. T.. (2020). The Development and Characterization of an scFv-Fc Fusion-Based Gene Therapy to Reduce the Psychostimulant Effects of Methamphetamine Abuse. J. Pharmacol. Exp. Ther. 374 (1), 16–23. doi: 10.1124/jpet.119.261180 32245884 PMC7289050

[B34] Holliger P. Hudson P. J. (2005). Engineered Antibody Fragments and the Rise of Single Domains. Nat. Biotechnol. 23 (9), 1126–1136. doi: 10.1038/nbt1142 16151406

[B35] Horn J. Friess W. (2018). Detection of Collapse and Crystallization of Saccharide, Protein, and Mannitol Formulations by Optical Fibers in Lyophilization. Front. Chem. 6, 4. doi: 10.3389/fchem.2018.00004 29435445 PMC5790775

[B36] Jäger V. Büssow K. Wagner A. Weber S. Hust M. Frenzel A.. (2013). High Level Transient Production of Recombinant Antibodies and Antibody Fusion Proteins in HEK293 Cells. BMC Biotechnol. 13:52. doi: 10.1186/1472-6750-13-52 23802841 PMC3699382

[B37] Jazayeri J. A. Carroll G. J. (2008). Fc-Based Cytokines. BioDrugs 22 (1), 11–26. doi: 10.2165/00063030-200822010-00002 18215087

[B38] Jiang S. Nail S. L. (1998). Effect of Process Conditions on Recovery of Protein Activity After Freezing and Freeze-Drying. Eur. J. Pharmaceut. Biopharmaceut. 45 (3), 249–257. doi: 10.1016/S0939-6411(98)00007-1 9653629

[B39] Kaneyoshi K. Uchiyama K. Onitsuka M. Yamano N. Koga Y. Omasa T. (2019). Analysis of Intracellular IgG Secretion in Chinese Hamster Ovary Cells to Improve IgG Production. J. Biosci. Bioeng. 127 (1), 107–113. doi: 10.1016/j.jbiosc.2018.06.018 30017708

[B40] Kaplon H. Reichert J. M. (2021). Antibodies to Watch in 2021. mAbs 13 (1), 1860476. doi: 10.1080/19420862.2020.1860476 33459118 PMC7833761

[B41] Kasper J. C. Friess W. (2011). The Freezing Step in Lyophilization: Physico-Chemical Fundamentals, Freezing Methods and Consequences on Process Performance and Quality Attributes of Biopharmaceuticals. Eur. J. Pharmaceut. Biopharmaceut. 78 (2), 248–263. doi: 10.1016/j.ejpb.2011.03.010 21426937

[B42] Kenanova V. Olafsen T. Crow D. M. Sundaresan G. Subbarayan M. Carter N. H.. (2005). Tailoring the Pharmacokinetics and Positron Emission Tomography Imaging Properties of Anti-Carcinoembryonic Antigen Single-Chain Fv-Fc Antibody Fragments. Cancer Res. 65 (2), 622–631. 15695407 PMC4154799

[B43] Kolhe P. Amend E. Singh S. K. (2010). Impact of Freezing on pH of Buffered Solutions and Consequences for Monoclonal Antibody Aggregation. Biotechnol. Prog. 26 (3), 727–733. doi: 10.1002/btpr.377 20039442

[B44] Kügler J. Wilke S. Meier D. Tomszak F. Frenzel A. Schirrmann T.. (2015). Generation and Analysis of the Improved Human HAL9/10 Antibody Phage Display Libraries. BMC Biotechnol. 15:10. doi: 10.1186/s12896-015-0125-0 25888378 PMC4352240

[B45] Latypov R. F. Hogan S. Lau H. Gadgil H. Liu D. (2012). Elucidation of Acid-Induced Unfolding and Aggregation of Human Immunoglobulin IgG1 and IgG2 Fc. J. Biol. Chem. 287 (2), 1381–1396. doi: 10.1074/jbc.M111.297697 22084250 PMC3256859

[B46] Lee T.-Y. Tjin Tham Sjin R. M. Movahedi S. Ahmed B. Pravda E. A. Lo K.-M.. (2008). Linking Antibody Fc Domain to Endostatin Significantly Improves Endostatin Half-Life and Efficacy. Clin. Cancer Res. An. Off. J. Am. Assoc. Cancer Res. 14 (5), 1487–1493. doi: 10.1158/1078-0432.CCR-07-1530 18316573

[B47] Lerner R. A. (2016). Combinatorial Antibody Libraries: New Advances, New Immunological Insights. Nat. Rev. Immunol. 16 (8), 498–508. doi: 10.1038/nri.2016.67 27374636

[B48] Li Z. Krippendorff B.-F. Sharma S. Walz A. C. Lavé T. Shah D. K. (2016). Influence of Molecular Size on Tissue Distribution of Antibody Fragments. mAbs 8 (1), 113–119. doi: 10.1080/19420862.2015.1111497 26496429 PMC5040103

[B49] Liu H. Gaza-Bulseco G. Sun J. (2006). Characterization of the Stability of a Fully Human Monoclonal IgG After Prolonged Incubation at Elevated Temperature. J. Chromatograp. B Anal. Technol. Biomed. Life Sci. 837 (1-2), 35–43. doi: 10.1016/j.jchromb.2006.03.053 16644295

[B50] Manning M. C. Chou D. K. Murphy B. M. Payne R. W. Katayama D. S. (2010). Stability of Protein Pharmaceuticals: An Update. Pharm. Res. 27 (4), 544–575. doi: 10.1007/s11095-009-0045-6 20143256

[B51] Mason M. Sweeney B. Cain K. Stephens P. Sharfstein S. T. (2012). Identifying Bottlenecks in Transient and Stable Production of Recombinant Monoclonal-Antibody Sequence Variants in Chinese Hamster Ovary Cells. Biotechnol. Prog. 28 (3), 846–855. doi: 10.1002/btpr.1542 22467228 PMC3394691

[B52] McCafferty J. Griffiths A. D. Winter G. Chiswell D. J. (1990). Phage Antibodies: Filamentous Phage Displaying Antibody Variable Domains. Nature 348 (6301), 552–554. doi: 10.1038/348552a0 2247164

[B53] Mensink M. A. Frijlink H. W. van der Voort Maarschalk K. Hinrichs W. L. J. (2017). How Sugars Protect Proteins in the Solid State and During Drying (Review): Mechanisms of Stabilization in Relation to Stress Conditions. Eur. J. Pharmaceut. Biopharmaceut. Off. J. Arbeitsgemeinschaft Fur. Pharmazeutische. Verfahrenstechnik. e.V. 114, 288–295. doi: 10.1016/j.ejpb.2017.01.024 28189621

[B54] Menzel C. Schirrmann T. Konthur Z. Jostock T. Dübel S. (2008). Human Antibody RNase Fusion Protein Targeting CD30+ Lymphomas. Blood 111 (7), 3830–3837. doi: 10.1182/blood-2007-04-082768 18230757

[B55] Milenic D. E. (2000). Radioimmunotherapy: Designer Molecules to Potentiate Effective Therapy. Semin. Radiat. Oncol. 10 (2), 139–155. doi: 10.1016/S1053-4296(00)80051-X 10727603

[B56] Moutel S. El Marjou A. Vielemeyer O. Nizak C. Benaroch P. Dübel S.. (2009). A Multi-Fc-Species System for Recombinant Antibody Production. BMC Biotechnol. 9, 14. doi: 10.1186/1472-6750-9-14 19245715 PMC2654441

[B57] Obrezanova O. Arnell A. de La Cuesta R. G. Berthelot M. E. Gallagher T. R. A. Zurdo J.. (2015). Aggregation Risk Prediction for Antibodies and Its Application to Biotherapeutic Development. mAbs 7 (2), 352–363. doi: 10.1080/19420862.2015.1007828 25760769 PMC4622581

[B58] Pace A. L. Wong R. L. Zhang Y. T. Kao Y.-H. Wang Y. J. (2013). Asparagine Deamidation Dependence on Buffer Type, Ph, and Temperature. J. Pharm. Sci. 102 (6), 1712–1723. doi: 10.1002/jps.23529 23568760

[B59] Patel S. M. Nail S. L. Pikal M. J. Geidobler R. Winter G. Hawe A.. (2017). Lyophilized Drug Product Cake Appearance: What Is Acceptable? J. Pharm. Sci. 106 (7), 1706–1721. doi: 10.1016/j.xphs.2017.03.014 28341598

[B60] Philo J. S. (2009). A Critical Review of Methods for Size Characterization of Non-Particulate Protein Aggregates. Curr. Pharm. Biotechnol. 10 (4), 359–372. doi: 10.2174/138920109788488815 19519411

[B61] Pikal-Cleland K. A. Carpenter J. F. (2001). Lyophilization-Induced Protein Denaturation in Phosphate Buffer Systems: Monomeric and Tetrameric Beta-Galactosidase. J. Pharm. Sci. 90 (9), 1255–1268. doi: 10.1002/jps.1078 11745778

[B62] Pikal-Cleland K. A. Rodríguez-Hornedo N. Amidon G. L. Carpenter J. F. (2000). Protein Denaturation During Freezing and Thawing in Phosphate Buffer Systems: Monomeric and Tetrameric Beta-Galactosidase. In Arch. Biochem. Biophys. 384 (2), 398–406. doi: 10.1006/abbi.2000.2088 11368330

[B63] Powers D. B. Amersdorfer P. Poul M.-A. Nielsen U. B. Shalaby M. Adams G. P.. (2001). Expression of Single-Chain Fv-Fc Fusions in Pichia Pastoris. J. Immunol. Methods 251 (1-2), 123–135. doi: 10.1016/S0022-1759(00)00290-8 11292488

[B64] Prestrelski S. J. Tedeschi N. Arakawa T. Carpenter J. F. (1993). Dehydration-Induced Conformational Transitions in Proteins and Their Inhibition by Stabilizers. Biophys. J. 65 (2), 661–671. doi: 10.1016/S0006-3495(93)81120-2 7693001 PMC1225768

[B65] Rhodes K. J. Trimmer J. S. (2006). Antibodies as Valuable Neuroscience Research Tools *Versus* Reagents of Mass Distraction. J. Neurosci. 26 (31), 8017–8020. doi: 10.1523/JNEUROSCI.2728-06.2006 16885215 PMC6673789

[B66] Roos Y. H. (1997). Frozen State Transitions in Relation to Freeze Drying. J. Thermal. Anal. 48 (3), 535–544. doi: 10.1007/BF01979500

[B67] Röthlisberger D. Honegger A. Plückthun A. (2005). Domain Interactions in the Fab Fragment: A Comparative Evaluation of the Single-Chain Fv and Fab Format Engineered With Variable Domains of Different Stability. J. Mol. Biol. 347 (4), 773–789. doi: 10.1016/j.jmb.2005.01.053 15769469

[B68] Russo G. Theisen U. Fahr W. Helmsing S. Hust M. Köster R. W.. (2018). Sequence Defined Antibodies Improve the Detection of Cadherin 2 (N-Cadherin) During Zebrafish Development. New Biotechnol. 45, 98–112. doi: 10.1016/j.nbt.2017.12.008 29289749

[B69] Russo G. Unkauf T. Meier D. Wenzel E. Wiesner R. Bischoff R.. In Vitro Evolution of Myc Tag Antibodies: In Depth Specificity and Affinity Analysis of Myc1-9E10 and Hyper-Myc, under revision. 10.1515/hsz-2021-0405 35312243

[B70] Ryu S.-I. Kim B.-G. Park M.-S. Lee Y.-B. Lee S.-B. (2007). Evaluation of Enhanced Hygroscopicity, Bifidogenicity, and Anticariogenicity of Enzymatically Synthesized Beta-Galactosyl-Trehalose Oligosaccharides. J. Agric. Food Chem. 55 (10), 4184–4188. doi: 10.1021/jf0636115 17429983

[B71] Sarciaux J. M. Mansour S. Hageman M. J. Nail S. L. (1999). Effects of Buffer Composition and Processing Conditions on Aggregation of Bovine IgG During Freeze-Drying. J. Pharm. Sci. 88 (12), 1354–1361. doi: 10.1021/js980383n 10585234

[B72] Schirrmann T. Büssow K. (2010). “Transient Production of scFv-Fc Fusion Proteins in Mammalian Cells,” in Antibody Engineering. Eds. Kontermann R. Dübel S. (Berlin, Heidelberg: Springer Berlin Heidelberg), 387–398.

[B73] Schmiedl A. Breitling F. Dübel S. (2000). Expression of a Bispecific dsFv-Dsfv’ Antibody Fragment in Escherichia Coli. Protein Eng. 13 (10), 725–734. doi: 10.1093/protein/13.10.725 11112512

[B74] Schwegman J. J. Hardwick L. M. Akers M. J. (2005). Practical Formulation and Process Development of Freeze-Dried Products. Pharm. Dev. Technol. 10 (2), 151–173. doi: 10.1081/pdt-56308 15926665

[B75] Singla A. Bansal R. Joshi V. Rathore A. S. (2016). Aggregation Kinetics for IgG1-Based Monoclonal Antibody Therapeutics. AAPS J. 18 (3), 689–702. doi: 10.1208/s12248-016-9887-0 26902302 PMC5256606

[B76] Skrabanja A. T. P. de Meere A. L. J. de Ruiter R. A. van den Oetelaar P. J. M. (1994). Lyophilisation of Biotechnology Products. J. Pharm. Sci. 48 (6), 311–317. 7850454

[B77] Sokolowska-Wedzina A. Chodaczek G. Chudzian J. Borek A. Zakrzewska M. Otlewski J. (2017). High-Affinity Internalizing Human scFv-Fc Antibody for Targeting FGFR1-Overexpressing Lung Cancer. Mol. Cancer Res. MCR 15 (8), 1040–1050. doi: 10.1158/1541-7786.MCR-16-0136 28483948

[B78] Steinwand M. Droste P. Frenzel A. Hust M. Dübel S. Schirrmann T. (2014). The Influence of Antibody Fragment Format on Phage Display Based Affinity Maturation of IgG. mAbs 6 (1), 204–218. doi: 10.4161/mabs.27227 24262918 PMC3929444

[B79] Tang X. Pikal M. J. (2004). Design of Freeze-Drying Processes for Pharmaceuticals: Practical Advice. Pharm. Res. 21 (2), 191–200. doi: 10.1023/b:pham.0000016234.73023.75 15032301

[B80] Thorat A. A. Munjal B. Geders T. W. Suryanarayanan R. (2020). Freezing-Induced Protein Aggregation - Role of pH Shift and Potential Mitigation Strategies. J. Controlled Release. Off. J. Controlled Release. Soc. 323, 591–599. doi: 10.1016/j.jconrel.2020.04.033 32335158

[B81] Thorat A. A. Suryanarayanan R. (2019). Characterization of Phosphate Buffered Saline (PBS) in Frozen State and After Freeze-Drying. Pharm. Res. 36 (7), 98. doi: 10.1007/s11095-019-2619-2 31087169

[B82] Thurber G. M. Schmidt M. M. Wittrup K. D. (2008). Antibody Tumor Penetration: Transport Opposed by Systemic and Antigen-Mediated Clearance. Adv. Drug Deliv. Rev. 60 (12), 1421–1434. doi: 10.1016/j.addr.2008.04.012 18541331 PMC2820307

[B83] Trivedi M. V. Laurence J. S. Siahaan T. J. (2009). The Role of Thiols and Disulfides on Protein Stability. Curr. Protein Pept. Sci. 10 (6), 614–625. doi: 10.2174/138920309789630534 19538140 PMC3319691

[B84] Valldorf B. Hinz S. C. Russo G. Pekar L. Mohr L. Klemm J.. (2021). Antibody Display Technologies: Selecting the Cream of the Crop. Biol. Chem. doi: 10.1515/hsz-2020-0377 33759431

[B85] van den Berg L. Rose D. (1959). Effect of Freezing on the pH and Composition of Sodium and Potassium Phosphate Solutions: The Reciprocal System KH2PO4-Na2HPO4-H2O. Arch. Biochem. Biophys. 81 (2), 319–329. doi: 10.1016/0003-9861(59)90209-7 13637993

[B86] Varshney D. Singh M. (2015). “History of Lyophilization,” in Lyophilized Biologics and Vaccines, vol. 24. Eds. Varshney D. Singh M. (New York, NY: Springer New York), 3–10.

[B87] Vromans H. van Laarhoven J. A. H. (1992). A Study on Water Permeation Through Rubber Closures of Injection Vials. Int. J. Pharmaceut. 79 (1-3), 301–308. doi: 10.1016/0378-5173(92)90122-I

[B88] Wang W. Nema S. Teagarden D. (2010). Protein Aggregation—Pathways and Influencing Factors. In Int. J. Pharmaceut. 390 (2), 89–99. doi: 10.1016/j.ijpharm.2010.02.025 20188160

[B89] Wang W. Singh S. Zeng D. L. King K. Nema S. (2007). Antibody Structure, Instability, and Formulation. J. Pharm. Sci. 96 (1), 1–26. doi: 10.1002/jps.20727 16998873

[B90] Wan L. Zhu S. Zhu J. Yang H. Li S. Li Y.. (2013). Production and Characterization of a CD25-Specific scFv-Fc Antibody Secreted From Pichia Pastoris. Appl. Microbiol. Biotechnol. 97 (9), 3855–3863. doi: 10.1007/s00253-012-4632-9 23250227

[B91] Wenzel E. V. Bosnak M. Tierney R. Schubert M. Brown J. Dübel S.. (2020). Human Antibodies Neutralizing Diphtheria Toxin *In Vitro* and *In Vivo* . Sci. Rep. 10 (1), 571. doi: 10.1038/s41598-019-57103-5 31953428 PMC6969050

[B92] Xu Y. Wang D. Mason B. Rossomando T. Li N. Liu D.. (2019). Structure, Heterogeneity and Developability Assessment of Therapeutic Antibodies. mAbs 11 (2), 239–264. doi: 10.1080/19420862.2018.1553476 30543482 PMC6380400

[B93] Yamauchi S. Kobashigawa Y. Fukuda N. Teramoto M. Toyota Y. Liu C.. (2019). Cyclization of Single-Chain Fv Antibodies Markedly Suppressed Their Characteristic Aggregation Mediated by Inter-Chain VH-VL Interactions. Mol. (Basel. Switzerland). 24 (14), 2620. doi: 10.3390/molecules24142620 PMC6681014 31323851

[B94] Zhang K. Geddie M. L. Kohli N. Kornaga T. Kirpotin D. B. Jiao Y.. (2015). Comprehensive Optimization of a Single-Chain Variable Domain Antibody Fragment as a Targeting Ligand for a Cytotoxic Nanoparticle. mAbs 7 (1), 42–52. doi: 10.4161/19420862.2014.985933 25484041 PMC4622481

[B95] Zhao J.-X. Yang L. Gu Z.-N. Chen H.-Q. Tian F.-W. Chen Y.-Q.. (2010). Stabilization of the Single-Chain Fragment Variable by an Interdomain Disulfide Bond and its Effect on Antibody Affinity. Int. J. Mol. Sci. 12 (1), 1–11. doi: 10.3390/ijms12010001 21339972 PMC3039938

