# Supplementary figures and images for: Shelf-Life Extension of Fc-Fused Single Chain Fragment Variable Antibodies by Lyophilization
Source: Front Cell Infect Microbiol. 2021 Nov 15;11:717689. doi: 10.3389/fcimb.2021.717689 (PMC8634725; doi:10.3389/fcimb.2021.717689)

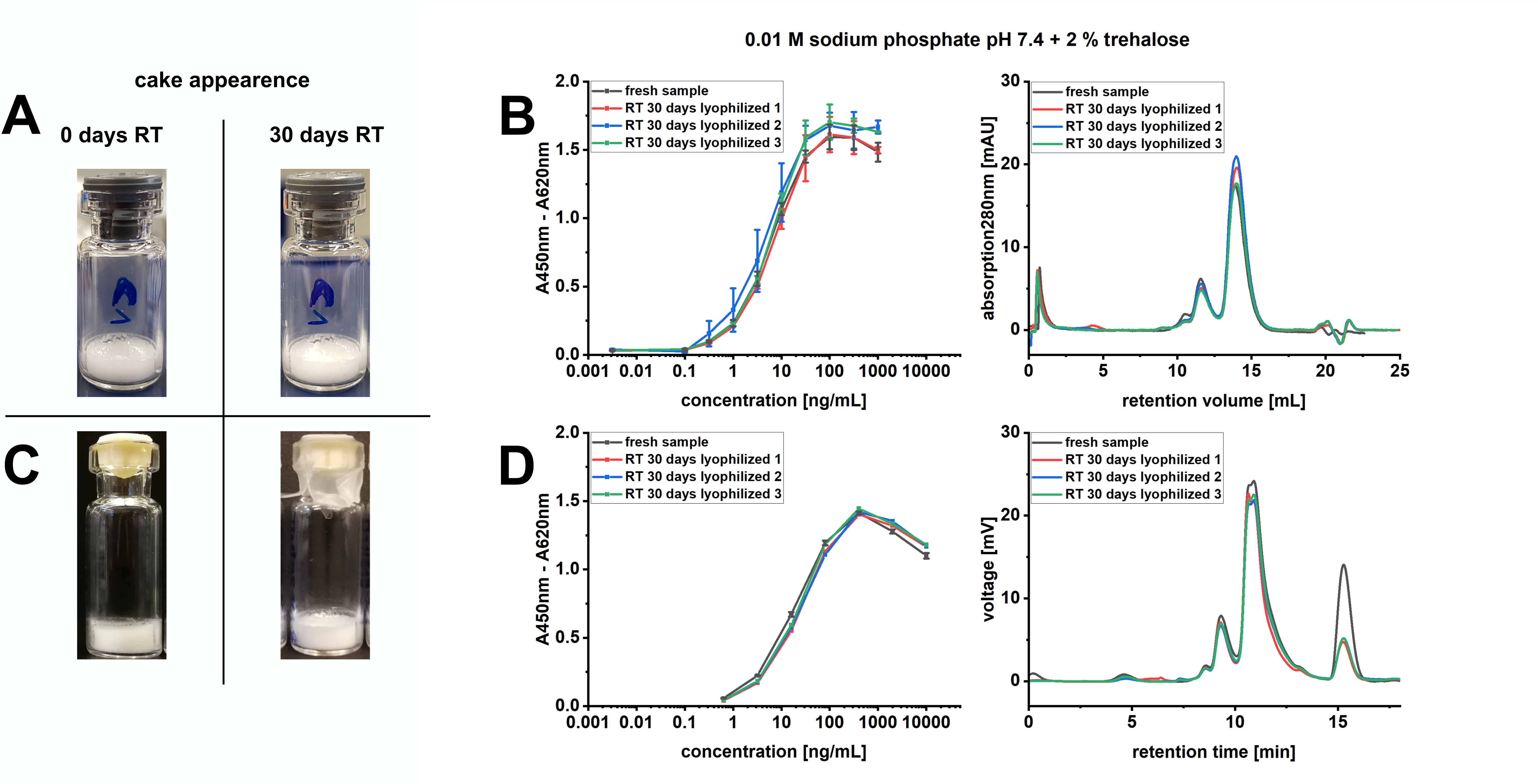

Supplement: Supplementary Figure 1 — Different glass vials size lyophilization comparison test. (A) Cake appearance immediately post lyophilization or after 30 days RT storage of 315 µL 0.5 mg/mL TUN219-2C1-hFc Ab solution in a cylindrical glass vial having a base-diameter of 15 mm and (C) 189 µL of the same material in a glass vial with 11 mm base-diameter. (B) Titration ELISA and SEC or SE-HPLC chromatogram of Ab lyophilized in 15 mm vials or (D) 11 mm vials, respectively. Each lyophilized Ab is compared, after reconstitution, to fresh material. [file Image_1.jpeg]
