# Supplementary material for: Shelf-Life Extension of Fc-Fused Single Chain Fragment Variable Antibodies by Lyophilization
Source: Front Cell Infect Microbiol. 2021 Nov 15;11:717689. doi: 10.3389/fcimb.2021.717689 (PMC8634725; doi:10.3389/fcimb.2021.717689)
Supplement: Supplementary Table 1 — Summary of EC50 values and percentages of monomers after lyophilization of TUN219-2C1-hFc in MTPs using different formulations. Values for lyophilizates are the mean of 3 individually lyophilized samples. Values of formulations that were either excluded by previous selection criteria or have not been in the scope of this study were not determined (“n.d.”). [file DataSheet_1.docx]

Supplemental Tables:

Supplement Table 1: Summary of EC_50_ values and percentages of monomers after lyophilization of TUN219-2C1-hFc in MTPs using different formulations. Values for lyophilizates are the mean of 3 individually lyophilized samples. Values of formulations that were either excluded by previous selection criteria or have not been in the scope of this study were not determined (“n.d.”).

Supplement Table 2: Summary of EC_50_ values and percentages of monomers after lyophilization of TUN219-2C1-hFc in 2 mL (15 mm) glass vials using different formulations and subsequent storage for 30 days. Values for lyophilizates are the mean of 3 individually lyophilized samples. Values of formulations that were either excluded by previous selection criteria or have not been in the scope of this study were not determined (“n.d.”).

Supplement Table 3: Summary of EC_50_ values and percentages of monomers after storage of VIF137-E7-hFc and SH1352-G9-hFc. “n.s.b.” refers to non-sigmoidal binding curves.
